# Combination of serum lipids and cancer antigens as a novel marker for colon cancer diagnosis

**DOI:** 10.1186/s12944-018-0911-5

**Published:** 2018-11-20

**Authors:** Tong Li, Yinfen Qian, Hongling Li, Jiusheng Deng

**Affiliations:** 1 Department of Endoscopy, Guizhou Provincial People’s Hospital, Zhongshandong Road 83, Guiyang, 550002 China; 2 Depatment of Cardiology, Wudan People’s Hospital, Guiyang, 550002 China; 3 Department of Pathology and Laboratory Medicine, Winship Cancer Institute, Emory University, Atlanta, 30322 USA

**Keywords:** Colon cancer, Serum lipids, Cancer antigens, Diagnostic marker

## Abstract

**Background:**

Colon cancer is a malignancy of the large intestine with high mortality and economic burden. Recent studies reveal a new relationship between blood lipids and the risk of cancer. The presents study aims to investigate the combination of serum lipids with cancer antigens as a novel diagnostic marker for colon cancer.

**Methods:**

Two hundred of colon cancer patients or healthy subjects were recruited. Serum lipids and cancer antigens such as total cholesterol (TC), high-density lipoprotein (HDL), carcinoembryonic antigen (CEA) and carbohydrate antigen 19–9 (CA19–9) were measured.

**Results:**

There were significantly lower level of serum TC or HDL, and significantly higher level of serum CEA or CA19–9 in patients than in healthy subjects. Serum TC or HDL in patients with advanced colon cancer was significantly lower than the ones with early stage disease. The level of serum TC or HDL in patients after surgical removal of colon cancer was significantly higher compared to the ones before surgery, but serum CEA or CA19–9 after surgery was significantly reduced in comparison with the ones before surgery. Combined TC, HDL, CEA and CA19–9 as a diagnostic marker for colon cancer had the highest positive predictive rate in comparison with individual, two or three of the parameters.

**Conclusions:**

The combination of serum TC, HDL, CEA and CA19–9 can be used as an effective marker for colon cancer, and offers a novel strategy for clinical diagnosis and monitoring the disease.

## Introduction

Colon cancer is a malignancy of the large intestine with high mortality and economic burden [1]. Most patients diagnosed with colon cancer are older than 50 years of age. Early diagnosis is the most effective way to prevent colon cancer, which help identify and remove polyps before turning into cancer. However, there are usually no symptoms in the early stage of the disease, and the proportion of patients with early diagnosis is low [2]. Thus, exploring high-predictive markers for the early diagnosis of colon cancer has potent clinical implication on the prevention and the treatment of the disease.

There are several methods for colon cancer diagnosis including blood-based test [3], colonoscopy [4] and barium meal imaging [5]. Blood test profiles the diagnostic biomarkers such as serum cancer antigens [6], circulating tumor cells [7], and cell-free DNA or RNA [8]. Carcinoembryonic antigen (CEA) [6] and carbohydrate antigen 19–9 (CA 19–9) [9] are the two most-used cancer antigens for monitoring colon cancer [10]. Circulating tumor cells originate from the primary or metastatic sites of colon cancer, and can directly reveal the status of the disease, however, the number of circulating tumor cells is very low [11]. DNA and RNA-based molecular method is more sensitive and useful than circulating tumor cells for the investigation of molecular heterogeneity and clonal divergence of colon cancer; but those molecular markers are still not utilized in clinic. Colonoscopy is a great tool for the diagnosis and the treatment of early colon cancer; however, patients often feel dis-comfortable with the procedure, which limits its early diagnostic value [12]. Recent studies demonstrate circulating lipids as potential markers [13, 14] and therapeutic targets [15] for colon cancer. Here, we discovered the combination of cancer antigens and serum lipids as an effective noninvasive diagnostic marker for colon cancer.

## Materials and methods

### Human subjects

Two hundred colon cancer patients without taking lipid-lowering medicines, and two hundred healthy subjects were recruited from January 2015 to December 2017. Patients were divided into four stages based on TNM staging system from the American Joint Committee on Cancer [16]: 28 patients in stage I, 62 in stage II, 58 in stage III, and 52 in stage IV. Healthy controls were under clinical examination including electrocardiograph using CARDIOFAX Multi-lead ECG Analyzer (ECG-2300).

(Shanghai Optical Medical Instrument Co., Ltd., China), cardiac ultrasound with the PHILIPS iE33 Ultrasound Machine (Philips, USA), chest X-ray with United Imaging UDR 770i X-Ray Scanner (Shanghai Lianying Medical Technology Co., Ltd., China), and blood test to exclude heart, lung, liver or kidney disease. The baseline characteristics of human subjects and the comparison of the baseline data between healthy controls and patients are listed in Table 1. The recruitment of human subjects in this study was approved by the Research Ethics Committee of Guizhou Provincial People’s Hospital.

**Table 1:** Characteristics of human subjects

Items	Healthy controls	Patients	*P* value
Number	200	200	/
Gender (Male/female)	120/80	114/86	0.864
Age	74.2 ± 9.3	73.8 ± 9.5	0.879
Body weight	59.8 ± 11.8	54.3 ± 14.2	0.595
RBC (10^12^/L)	4.6 ± 0.5	4.5 ± 0.6	0.323
Hb (g/L)	137.4 ± 14.5	134.2 ± 19.8	0.435
HCT	0.42 ± 0.03	0.4 ± 0.04	0.440
ALT (U/L)	54.6 ± 24.1	59.4 ± 36.1	0.510
TBIL (μmol/L)	23.7 ± 12.2	27.2 ± 13.6	0.312
ALB (g/L)	41.3 ± 6.4	41.2 ± 7.6	0.958
PT (second)	12.6 ± 1.1	12.5 ± 1.2	0.547
BUN (mmol/L)	4.8 ± 1.3	5.0 ± 1.4	0.395
CR (μmol/L)	69.0 ± 14.5	70.6 ± 16.4	0.614

*RBC* red blood cells, *Hb* Hemoglobin, *HCT* hematocrit, *ALT* alanine aminotransferase, *TBIL* total bilirubin, *ALB* albumin, *PT* prothrombin time, *BUN* Blood urea nitrogen, *CR* Serum creatinine

### Food supply for patients

Department of Diet Nutrition in the Hospital provided food for colon cancer patients during perioperative period. The food for each patient everyday consisted of 300 g of cereals, 500 g of vegetables (such as celery, leeks, cabbage, radish or other green leafy vegetables), 200 g of meat (such as fish, poultry, eggs or pork), 250 g of milk. Surgery often affect patients’ appetite leading to satiety, thus patients were also supplemented with oral vitamin B6. The scores of symptom on loss of appetite, bloating, nausea or early satiety in patients as well as in healthy controls were counted and listed in Table 2, following the criterion: no symptom, 0 point; weak or occasional occurrence, 1 point; strong or often occurrence, 2 points; painful, 3 points.

**Table 2:** Comparison of symptom scores between healthy controls and patients during perioperative period (*n* = 200)

Subjects	Loss of appetite	nausea	Early satiety	Bloating
Controls	0.51 ± 0.13	0.54 ± 0.43	0.82 ± 0.44	1.11 ± 0.51
Patients	1.78 ± 0.59^*^	1.46 ± 0.53^*^	2.10 ± 0.61*	1.69 ± 0.47^*^

*P < 0.05

### Blood collection

Peripheral blood (5 ml) was collected for blood test via elbow vein from patients when admission, before and after surgery (complete mesocolic excision to remove colon cancer), or when follow-up (One month after surgery), or from healthy controls. Additional 5 ml of fasting venous blood was also collected for the measurement of CEA and CA19–9. Serum was isolated from blood samples after centrifugation for 15 min at 3000 rpm at 4 °C, and stored at − 80 °C for use.

### Blood analyses

The baseline blood profile including red blood cells (RBC), Hemoglobin (Hb) and hematocrit (HCT) was analyzed on the Sysmex XN2000 Automatic Hematology Analyzer (Sysmex, USA). Blood prothrombin time (PT) was examined on the Sysmex-CA510 automatic coagulation analyzer machine (Sysmex, Japan) following the protocol from the company. Parameters of liver and kidney functions including alanine aminotransferase (ALT), total bilirubin (TBIL), albumin (ALB), blood urea nitrogen (BUN) and serum creatinine (CR) were analyzed using an Abbott ARCHITECT C16000 Automatic Biochemical Analyzer (Abbott, USA) following the instruction from the company.

### Quantification of serum lipids and cancer antigens

Quantification of serum lipids including total cholesterol (TC), triglyceride (TG), high-density lipoprotein (HDL), and low-density lipoprotein (LDL) was performed on an Abbott ARCHITECT C16000 Automatic Biochemical Analyzer (Abbott, USA) with kits provided by the company. Serum CEA and CA19–9 was quantified by enzyme-linked immunosorbent assay (ELISA) on the ES-300 Enzyme Immunoassay Analyzer (Boehringer, Germany) with kits purchased from Invitrogen (USA), following the instruction from the company. The clinical reference value of TC, HDL, CEA and CA19–9 were 3.3 mmol/L, 1.04 mmol/L, 6.9 U/ml, and 37 U/ml respectively.

### Statistical analysis

All data were analyzed using the SPSS 19.0 software package (SPSS, Inc., Chicago, IL, USA), and presented as mean ± SD (standard deviation). Student t-test was used to assess difference between patients and healthy controls. Pearson correlation technique was used to analyze the correlation between disease duration and serum TC or HDL change. The significance of the positive predictive rate was determined by chi-square test. *P* < 0.05 was considered as statistical significance.

## Results

### Decreased serum TC, HDL and increased CEA and CA19–9 in colon cancer patients

**Fig. 1:**
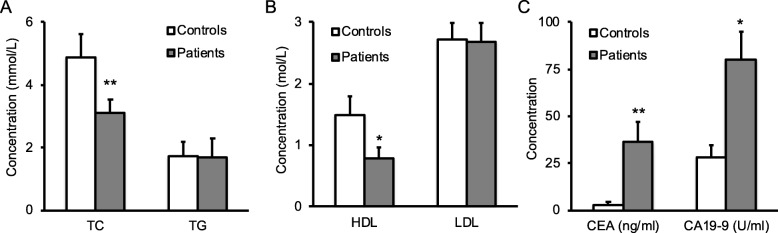
Serum lipids and cancer antigens in patients and healthy subjects. Blood samples collected from 200 colon cancer patients or 200 healthy controls. (**a**-**b**) Serum lipids including TC, TG (**a**), HDL and LDL (**b**) were quantified on an Automatic Biochemical Analyzer machine. (**c**) Serum CEA and CA19–9 were also measured by ELISA. Data were presented as mean ± SD, and analyzed by student t-test. *P < 0.05, **P < 0.01

**Fig. 2:**
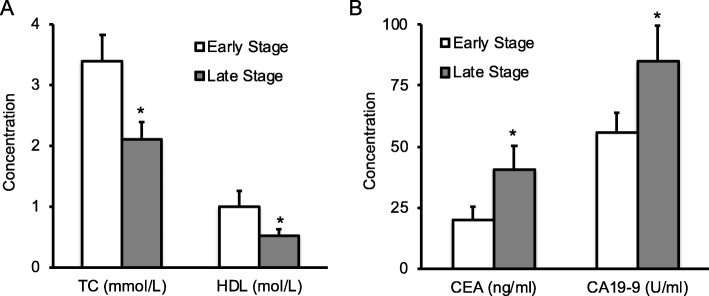
Serum lipids and cancer antigens in patients with early or late stage of colon cancer. Blood were collected from 90 patients with early stage of colon cancer and 110 patients with late stage of colon cancer. (**a**) Serum TC and HDL in blood samples were profiled on the Automatic Biochemical Analyzer machine. (**b**) Serum CEA and CA19–9 were quantified by ELISA. Data were presented as mean ± SD, and examined by student t-test. *P < 0.05

**Fig. 3:**
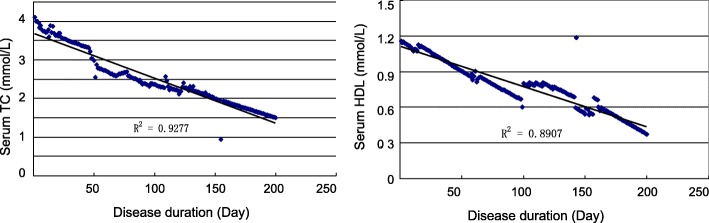
Correlation between the duration of disease and the change of serum TC or HDL in patients. The data of disease duration, serum TC or HDL were collected from colon cancer patients (*n* = 200), and subjected to Pearson correlation analysis. R^2^ is the coefficient of determination

To examine the levels of lipids in elderly patients with colon cancer, we analyzed serum levels of TG, TC, HDL and LDL in healthy subjects and two hundred patients without any treatment. Statistical analysis demonstrated that patients had significantly lower levels of serum TC (Fig. 1a) and HDL (Fig. 1b) than control group; however, there was no significant difference on serum TG (Fig. 1a) and LDL (Fig. 1b). We next analyzed the levels of CEA and CA19–9, and found that patients had high levels of serum CEA and CA19–9 compared to healthy controls (Fig. 1c). Colon cancer has four stages of disease development based on its invasion, lymph node involvement and metastasis. Stage I and II are the early stage; stages III and IV are the late or advanced stage. To further examine the change of serum TC, HDL, CEA and CA19–9 during colon cancer development, we compared the patients in the early stage (I and II) with the ones in the late stage (III and IV), and found that patients with the late stage of colon cancer had significantly lower levels of serum TC and HDL (Fig. 2a), but had significantly higher levels of serum CEA and CA19–9 (Fig. 2b). Further analyses of the correlation between the duration of disease and serum TC or HDL change revealed that there was a strong inverse correlation between disease duration and the change of serum TC or HDL in colon cancer patients (Fig. 3).

### Increased serum TC, HDL and decreased CEA and CA19–9 in treated patients

**Fig. 4:**
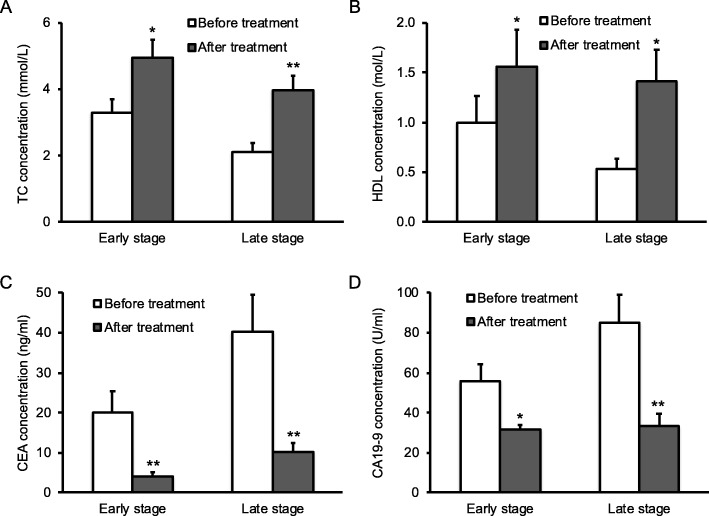
Serum lipids and cancer antigens in colon cancer patients before and after surgery. Blood samples were harvested from 200 patients before and after complete mesocolic excision of colon cancer from patients. Serum TC (**a**), HDL (**b**), CEA (**c**) and CA19–9 (**d**) were analyzed on an Automatic Biochemical Analyzer (A and B) or quantified by ELISA (**c** and **d**). Data were presented as mean ± SD, and tested by student t-test. *P < 0.05. **P < 0.01

To test our hypothesis that complete mesocolic excision of colon cancer by surgery could affect the levels of TC, HDL and cancer antigens in patients, we analyzed serum TC, HDL, CEA and CA19–9 before and after surgery respectively. Statistical analysis showed that surgical treatment significantly enhanced serum TC (Fig. 4a) and HDL (Fig. 4b) in both groups of patients with early or late stage of the disease, up to the levels similar to the ones in healthy controls (Fig. 1a and b). Moreover, surgical removal of colon cancer markedly reduced serum CEA (Fig. 4c) and CA19–9 (Fig. 4d) in patients with early or late stage of the disease, down to the levels similar to the ones in healthy controls (Fig. 1c).

### A combination of serum TC, HDL, CEA and CA19–9 enhanced positive predictive rate for colon cancer

To test our hypothesis that a combination of serum TC, HDL, CEA and CA19–9 could increase the positive predictive rate of colon cancer, we combined the four parameters, or three or two of the four parameters as a complex diagnostic marker for colon cancer. We discovered that the combination of TC, HDL, CEA and CA19–9 parameters significantly enhanced the positive predictive rate for colon cancer diagnosis in patients, with the highest rate 87.5% (Table 3) in comparison with the combination of three or two of the four parameters respectively; although the combination of three parameters had significantly higher positive predictive rate than the combination of two parameters, and the latter also had higher predictive rate than individual parameter (Table 3).

**Table 3:** Predictive rate of diagnostic marker(s) for colon cancer patients

Diagnostic marker(s)	Positive cases	Predictive rate (%)	Significance
TC	39	19.5	/
HDL	26	13.5	/
CEA	78	39.0	/
CA19–9	60	30.0	/
TC + CEA	97	48.5	*P < 0.05*
TC + CA19–9	94	47.0	*P < 0.05*
HDL + CEA	92	46.0	*P < 0.05*
HDL + CA19–9	95	47.5	*P < 0.05*
TC + HDL + CEA	120	60.0 ^a^	*P < 0.05*
TC + HDL + CA19–9	116	58.0 ^a^	*P < 0.05*
TC + CEA + CA19–9	150	75.5 ^a^	*P < 0.05*
HDL + CEA + CA19–9	144	72.0 ^a^	*P < 0.05*
TC+ HDL + CEA + CA19–9	175	87.5 ^a b^	*P < 0.01*

^a^Compared with the combination of two diagnostic markers

^b^ Compared with the combination of three diagnostic markers

## Discussion

In this study, we investigated the diagnostic value of serum lipids and cancer antigens for colon cancer, and demonstrated for the first time that the combination of serum TC, HDL, CEA and CA19–9 markedly enhanced the positive predictive rate of colon cancer.

Serum lipids including TC, TG, LDL and HDL are the biomarkers for cardiovascular diseases (CVD). High TC, TG, LDL and low HDL are a well-established risk profile for CVD, in particular for coronary heart diseases. However, recent studies reveal a new relationship between blood lipids and the risk of the most common cancer entities. For example, low levels of serum lipids is positively associated with cancer and its mortality [13, 14, 17]. High level of TC or HDL is associated with a reduction of cancer mortality [18]. In this study, we found that serum level of TC or HDL in colon cancer patients was significantly lower than those in healthy controls; moreover, serum level of TC or HDL in the late stage of colon cancer was markedly lower than those in the early stage, suggesting the abnormal levels of serum TC and HDL are the risk factors and potential biomarkers for colon cancer.

The possible effects of low level of lipids and lipoproteins on cancer include the increase of tumor angiogenesis, the disturbance of host immune system; low level of circulating anti-oxidants, the alteration of protein function, the decrease of tumor apoptosis and the increase of tumor cell proliferation [19–24]. Proto-oncogenes and cell proliferation genes are involved in tumor development. Abnormal lipid metabolism is found to correlate with proto-oncogene activation and tumor cell proliferation [25]. Cholesterol induces autophagic and apoptotic death in gastric carcinoma cells [26], suggesting hypocholesterolemia increases the mortality of cancer patients. In our study, we found that the decrease of serum TC and HDL is consistent with the development of colon cancer in elderly patients; surgical removal of colon cancer significantly enhances the levels of serum TC and HDL, further indicating both TC and HDL are potential biomarkers for monitoring the development and the treatment of colon cancer. The mechanisms underlying the association of low serum lipids and lipoproteins with colon cancer are not fully understood. Lipids are the main energy source and key components of colon cancer cell membrane and crucial signaling molecules on the membrane. Cancer cell proliferation enhances cholesterol anabolism with increased lipid synthesis and absorption by the cells, which could reduce serum cholesterol level including HDL and TC in colon cancer patients. Anorexia could also lead to hypolipidemia [18] in colon cancer patients. HDL has anti-inflammatory and antioxidant functions [27]. It is possible that reduced serum HDL could promote inflammation and oxidative stress involved in colon cancer development. In addition, reduced HDL is linked with insulin resistance [28], enhancing the risk of colon cancer.

Cancer antigens CEA and CA19–9 are the two most common colon cancer markers [29], used not only for preoperative assessment of extent and outcome of the disease, but also for monitoring recurrence after surgery. Increased serum concentrations of CEA and CA19–9 are associated with advanced invasion, lymph node metastasis, and short survival [30, 31]. However, CEA and CA19–9 are not high-valuable markers for the diagnosis of the early stage colon cancer [32–34]. In this study, we confirmed the elevation of serum CEA and CA19–9 in colon cancer patients; moreover, patients with the late stage colon cancer had significantly higher levels of both cancer antigens than the ones with the early stage disease. Considering the advantage of combined CEA and CA19–9 for colon cancer diagnosis [35], and the profile of serum TC and HDL in patients observed in this study, we assumed that the combination of cancer antigens and serum lipids could serve as a more effective marker for the diagnosis of colon cancer. Indeed, we found that the combination of CEA, CA19–9, TC and HDL gave the highest positive predictive rate (87.5%) of colon cancer in comparison with individual marker or the combination of two or three of the four markers. Our results suggest that the combination of serum lipids and cancer antigens could be utilized as a novel diagnostic marker for colon cancer.

## Conclusions

In conclusion, we discovered for the first time that the combination of serum TC, HDL, CEA and CA19–9 can serve as an effective marker for colon cancer, and offer a novel strategy for clinical diagnosis and monitoring the disease.

## Abbreviations

ALB: Albumin
ALT: Alanine aminotransferase
BUN: Blood urea nitrogen
CA19–9: Carbohydrate antigen 19–9
CEA: Carcinoembryonic antigen
CR: Creatinine
ELISA: Enzyme-linked immunosorbent assay
HDL: High-density lipoprotein
LDL: Low-density lipoprotein
PT: Prothrombin time
TBIL: Total bilirubin
TC: Total cholesterol
TG: Triglyceride
